# Nanocomposites of Opuntia ficus-indica mucilage-mediated selenium nanoparticles and nanochitosan as antidiabetic candidates in streptozocin-induced diabetic rats

**DOI:** 10.3389/fbioe.2026.1836736

**Published:** 2026-06-10

**Authors:** Haddad A. El Rabey, Sara E. Elnagar, Ayman Y. Allam, Amnah Obidan, Adel I. Alalawy, Fahad M. Almutairi, Ahmad A. Tayel

**Affiliations:** 1 Biochemistry Department, Faculty of Science, University of Tabuk, Tabuk, Saudi Arabia; 2 Department of Fish Processing and Biotechnology, Faculty of Aquatic and Fisheries Sciences, Kafrelsheikh University, Kafrelsheikh, Egypt; 3 Department of Food Science and Technology, Faculty of Agriculture, Menoufia University, Shibin El Kom, Egypt

**Keywords:** bioactivity, biopolymers, green synthesis, nanoconjugates, streptozocin-induced diabetes

## Abstract

**Background:**

Diabetes is considered a serious chronic disease that carries a high risk of significant complications. Recently, the utilization of natural hypoglycemic plants has been increasing for the management of diabetes due to their biosafety, low side effects, and economical features. *Opuntia ficus-indica* (OFI) fruit has various pharmacological properties as a source of bioactive compounds, which have shown effectiveness in diabetes treatment.

**Methods:**

Fruits peel mucilage (FM) was extracted from OFI fruit for the green synthesis of selenium nanoparticles (SeNPs), which were then encapsulated with chitosan (Ch) to generate nanocomposites (Ch/FM/SeNPs). This study was carried out to evaluate the antidiabetic activity of nanocomposites (Ch/FM/SeNPs) against streptozotocin (STZ)-induced diabetic rats. Accordingly, rats were divided into six groups, namely, normal control (GI), diabetic control (GII), treatment groups (GIII, GIV, and GV) administered nanocomposite powder (Ch/FM/SeNPs) with concentrations 0.1, 0.2, and 0.3 mg/kg, respectively, and (GVI) received glibenclamide at a dose of 5 mg/kg for 28 days. At the end of the treatment, the rats were subjected to analysis including body weight, blood glucose level, lipid profile, and hepatic and renal function tests.

**Results:**

The nanocomposite (Ch/FM/SeNPs) had an average particle size (PS) of 81.18 nm and a positive charge (+30.11 mV), with an equivalent ratio of Ch and FM/SeNPs (1:1). The average particle size of FM/SeNPs was 6.34 nm with a negative charge (−25.93 mV). The morphological characteristics indicated semi-spherical shapes of the nanocomposites (NCs) and stabilization and homogenous distribution of SeNPs within the polymer nanocomposites. The results indicated that treatment of diabetic rats with nanocomposite powder (Ch/FM/SeNPs) at a concentration of 0.3 mg/kg resulted in significant increases in body weight, along with significant reductions in blood glucose levels and improvement in biological parameters, lipid profile, and hepatic and renal function.

**Conclusion:**

Promisingly, the nanocomposite (Ch/FM/SeNPs) can be used as a hypoglycemic compound; therefore, it could be recommended for the development of antidiabetic drugs.

## 1 Introduction

Diabetes mellitus (DM) is one of the major non-communicable chronic public health issues. The metabolic malfunction of DM characterized by abnormally increased levels of blood glucose due to low insulin secretion from the dysfunction of pancreatic β-cells or impaired insulin uptake by cells because of resistance mechanisms, which result in significant morbidity and mortality (Roden and Shulman, 2019; Galicia-Garcia et al., 2020). In 2024, according to the International Diabetes Federation (IDF) data, the estimated number of people between the ages of 20 and 79 years with diabetes worldwide is approximately 589 million. By 2050, the total number of people with diabetes is projected to increase by 45% to reach 853 million.

Natural hypoglycemic plants have garnered increasing attention in recent decades due to their low cost, availability, and minimal side effects, making plant-based preparations the primary alternative among available therapies, especially in developing countries (Salehi et al., 2019; Hu et al., 2022). It is difficult to control DM without any side effects, so drugs derived from plants may play a significant role in DM treatment with fewer side effects (Guo et al., 2020; Kaliaperumal et al., 2024). Natural products derived from plant sources have been used for treatment and prevention of several pathologic diseases, including high blood pressure, DM, cancer, and heart disease (Vinayagam et al., 2017; Zhao et al., 2020).

*Opuntia ficus-indica* (OFI) fruit is a dicotyledonous angiosperm plant and member of the Cactaceae family native to tropical and subtropical regions that is widely distributed globally. OFI fruit possesses numerous pharmacological properties, including anti-bacterial, anti-oxidant, anti-inflammatory, anti-diabetic, and anti-tumor (Avila-Nava et al., 2014; Martins et al., 2023; Besné-Eseverri et al., 2026). OFI fruit peels have a high antioxidant capacity, which shows strong antioxidative properties attributed to their ability to scavenge free radicals due to the presence of natural antioxidants, such as fatty acids, phenolic compounds, organic acids, and flavonoids (Chougui et al., 2013; Abbas et al., 2022). According to recent research, the flavonoids present in OFI fruit are effective in treating diabetes (Alqudah et al., 2024).

Elemental selenium nanoparticles (SeNPs) have attracted significant attention because of their distinctive biological activities, low or no toxicity, biocompatibility, high permeability, and intestinal absorption (Aly Khalil et al., 2024). Selenium (Se) is an essential micronutrient for a healthy life and has been reported to play a role in maintaining immunity, redox balance, and anti-cancer activity, as well as in diabetes, mental disorders, infections, and inflammation (Huang et al., 2020; Estarriaga-Navarro et al., 2026). Se supplementation has antidiabetic properties by decreasing plasma glucose levels in humans and diabetic rats at adequate concentrations (Lin et al., 2018; Steinbrenner et al., 2022). Additionally, Se is involved in the synthesis of different selenoproteins, such as glutathione peroxidase, and has been proven to improve the survival of pancreatic β-cells and insulin secretion (Meng et al., 2017; Moon and Oh, 2023).

Various studies show that polysaccharides conjugated with Se can enhance biological functions and address relevant drawbacks. Natural polysaccharide-based nanoparticles/nanometals can encapsulate therapeutic compounds and extend their duration of *in vivo* retention. Chitosan (Ch), a natural polysaccharide, is widely used in nanomedicine and advanced drug delivery technologies due to its low toxicity, biocompatibility, reduced pharmacological side effects, and capacity to improve drug bioavailability. Regarding diabetes and metabolic disorders, Ch has been shown to reduce hyperglycemia, protect pancreatic β-cells, and modulate lipid metabolism (Yang et al., 2023; Abdella et al., 2026).

The study aims to investigate a novel therapeutic strategy that may offer cost-effectiveness compared to current treatments, potentially reducing the economic burden associated with the management of diabetes. In this study, mucilage was extracted from OFI fruit for the green synthesis of Se NPs, which were then encapsulated with Ch for characterization and to investigate the nanocomposite (Ch/FM/SeNPs) effects in a streptozotocin (STZ)-induced DM rat model. Additionally, biochemical examination was conducted for further assessment of the findings.

## 2 Materials and methods

### 2.1 Mucilage extraction

The extraction process was carried out from Opuntia ficus-indica (OFI) fruits, which were purchased from a local market in Kafr El-Sheikh, Cairo, Egypt. The methodology extraction was slightly adjusted from a recent procedure (Aborabu et al., 2024). The fruits were carefully cleaned to remove impurities from the fruit surface and then peeled, followed by immersion of the peels in double-distilled water (DW) at a concentration of 1:30 (*w*/*v*) ratio and mechanically stirring using a magnetic stirrer at room temperature (RT; 25 °C ± 1 °C) for 6 h. A double-layer cloth filter was used to separate the fruit mucilage (FM) from the peel fruit residues. The filtrate was mixed with an equal volume of 96% ethanol for FM precipitation, and the mixture was maintained at a low temperature (4 °C ± 1 °C) for 2 h. The obtained mucilage was separated by centrifugation (4,650 × g) and then freeze-dried.

### 2.2 Biosynthesis of FM/SeNPs

The protocol for SeNP mediation/bioreduction using FM was adjusted from a recent study (Shehab et al., 2022). Initially, a fresh solution of Na_2_SeO_3_ (sodium selenite, 0.01%, *w*/*v*) in DW was prepared. Thereafter, 15 mL of this solution was mixed with an equal *v*olume of FM solution (1%, *w*/*v*) and stirred at 610 × g for 95 min. To accelerate the biosynthesis of SeNPs, 5 mL of ascorbic acid solution (1.0% w/v) was added gradually to the mixture. A color change of the solution was observed visually, shifting to brownish orange, indicating the reduction of Se ions and the formation of FM-SeNPs. The formed FM-SeNPs were collected by centrifugation at 9,800 × g for 35 min (2–16KL, Sigma; Osterode am Harz, Germany), then washed with DW and re-centrifuged to remove any excess materials, and freeze-dried. This process was carried out five times to obtain pure SeNPs, primarily to remove associated FM particles.

### 2.3 Preparation of nanocomposites (Ch/FM/SeNPs)

Solutions of 1% (*w*/*v*) were prepared from Ch in acetic acid (1.5%, *v*/*v*) and FM/SeNPs in DW; the solutions were sonicated individually for 15 min. Then, 20 mL of FM/SeNPs solution was gently dropped into Ch solution under vigorous stirring (745 × g) for the formation of nanocomposites (Ch/FM/SeNPs), with three trails prepared at different concentrations:

- T_1_ (2 FM/SeNPs: 1 Ch)
- T_2_ (1 FM/SeNPs: 1 Ch)
- T_3_ (1 FM/SeNPs: 2 Ch)

The solutions were further stirred for an additional 40 min after complete addition. The nanocomposite (NC) particles were collected *via* centrifugation (10,520 × g), washed, re-centrifuged, and dried. The dry powder was collected, stored in airtight bottles, and kept in a refrigerator at −4 °C until use.

### 2.4 Characterization of NPs

#### 2.4.1 Spectroscopy analysis using Fourier-transform infrared spectroscopy

Fourier-transform infrared (FT-IR) spectroscopy was used to analyze the biochemical bonds/functional groups in the investigated compounds and evaluate their interactions present in the NCs, including FM, FM/SeNPs, Ch, and Ch/FM/SeNPs. After mixing the compounds/NC powders with KBr, FT-IR spectra (JASCO FT-IR-360, Tokyo, Japan) were recorded. The IR spectra (transmission mode) were measured over a wavenumber range of 4,000–450 cm^−1^.

#### 2.4.2 Particle size and charge assessment of NPs

The fabricated nanomaterials/NCs were dissolved in DW and sonicated for 10 min at 44 W. Then, the particle size (PS) distribution, charge, and zeta (ζ) potential were determined at RT using a Zetasizer (Malvern™, Worcestershire, United Kingdom) *via* the dynamic light scattering (DLS) technique. The polydispersity index (PDI) obtained from DLS quantifies the size uniformity of nanoparticles in solution.

#### 2.4.3 Transmission electron microscopy imaging

The structural features of biosynthesized SeNPs, such as size, distribution, apparent shape, and morphology, were evaluated by transmission electron microscopy (TEM) (JEOL JEM-2100, Japan; at an operating accelerating voltage of 200 kV).

#### 2.4.4 Scanning electron microscopy imaging

Nanocomposites (Ch/FM/SeNPs NCs; T_1_, T_2_, and T_3_) were characterized for surface morphology, structure, and size by scanning electron microscopy (SEM) imaging using a JEOL, JSM IT100 (Japan).

#### 2.4.5 ICP–MS examination of selenium contents

The diluted solution was filtered through a plastic tube designed for the autosampler of an ICP–MS Agilent Series instrument (Agilent-4500, Palo Alto, CA), which was used to analyze Se content. The analytical conditions were as follows: RF power of 1200 W, plasma gas (Ar) at 16.0 ml/min, auxiliary gas (Ar) at 1.0 ml/min, carrier gas (Ar) at 1.14 ml/min, a glass spray chamber, a Barbington-type nebulizer, sampling depth of 8.2 mm, Ni/Ni sampling cone/skimmer cone, and Se analysis at m/z 82. The concentrations of Se in the samples were determined from the regression line (r2 = 0.999) obtained using a standard selenium solution.

### 2.5 Experimental animals

All experimental protocols were approved by the Institutional Animal Care and Animal Ethics Committee, Faculty of Aquatic and Fisheries Sciences, Kafr El-Sheikh University, Egypt, and were conducted in accordance with the NIH Guidelines for the Care and Use of Laboratory Animals and EU Directive 2010/63 on the protection of animals used for scientific purposes.

The study experiments also adhered to the regulations and guidelines of ARRIVE (https://arriveguidelines.org/).

Wistar albino (WA) male rats (150 ± 5 g) were procured from a confined Animal House Colony and maintained in polyurethane cages under standard environmental conditions of temperature (25 °C ± 1 °C) and a 12/12 h light/dark cycle. They were provided with a standard rat diet and water *ad Libitum*. All animals were allowed a 12-day acclimatization period to adapt to the new housing conditions before being used in the study. The experimental animals (*n* = 36) were divided into six experimental groups, with six rats per group, as follows: GI (normal control group), which was administered DW orally; GII (diabetic control group), which received a single dose of STZ (50 mg/kg) and was administered DW orally; GIII, GIV, and GV were diabetic groups that received STZ and nanocomposite (Ch/FM/SeNPs) powder at concentrations 0.1, 0.2, and 0.3 mg/kg, respectively, for 28 days; and GVI was a diabetic group that received glibenclamide at a dose of 5 mg/kg for 28 days. All treatments were administered *via* oral gavage once daily at a fixed time for 28 consecutive days to minimize physiological variability.

- Group I: normal control (administered DW orally).
- Group II: diabetic control.
- Group III: diabetic + Ch/FM/SeNPs nanocomposite powder treatment (0.1 mg/kg).
- Group IV: diabetic + Ch/FM/SeNPs nanocomposite powder treatment (0.2 mg/kg).
- Group V: diabetic + Ch/FM/SeNPs nanocomposite powder treatment (0.3 mg/kg).
- Group VI: diabetic + glibenclamide (5 mg/kg).

### 2.6 Acute toxicity

A random sampling procedure was used to select six male WA rats (150 ± 5 g). Prior to the experiment, the animals were fasted for 4 h and maintained under standard temperature conditions (25 °C ± 1 °C). The rats were provided free access to water. The nanocomposite (Ch/FM/SeNPs) powder was dissolved in DW and administered orally by gavage at the initial doses. The general behavior of the experimental rats was observed continuously over a period of 24 h to detect any signs of toxicity and mortality.

### 2.7 Diabetes induction

After adaptation period, diabetes was induced in overnight-fasted animal models by an intraperitoneal injection of STZ (50 mg/kg b.w prepared in 0.1 M normal saline). The rats were provided with a 5% glucose solution in their feeding bottles for the following 24 h to prevent hypoglycemia after STZ injection. Blood glucose levels were recorded daily using a blood glucometer (Accu-Chek Active) by pricking the tip of the tail. Seventy-two hours after STZ administration, diabetes was confirmed in rats with fasting blood glucose levels *>*250 mg/dl, and body weight was monitored throughout the 28-day study.

### 2.8 Biochemical analysis

At the end of the treatment period, blood samples were collected *via* the retro-orbital plexus under mild ether anesthesia from overnight-fasted rats. Blood glucose levels were assessed, and the remaining blood was processed and preserved at −80 °C until used for biochemical analysis (Gobinath et al., 2022). The biochemical parameters, including lipid profile (total cholesterol, triglycerides, HDL, and LDL), liver function tests [serum alanine aminotransferase (ALT) and serum aspartate aminotransferase (AST)], and kidney function tests [serum creatinine and serum urea], were estimated from the collected serum samples. A Reflotron Plus analyzer (Roche, Germany) was used to measure these biochemical parameters (Parasuraman et al., 2019).

### 2.9 Statistical analysis

Fasting blood glucose and biochemical parameter values were expressed as the mean ± standard error of mean (S.E.M.) and analyzed using ANOVA and *post hoc* Dunnett’s *t*-test. A *p*-value *<*0.05 was considered statistically significant between the groups.

## 3 Results and discussion

### 3.1 Optical assessments of FM-biosynthesized SeNPs

**FIGURE 1:**
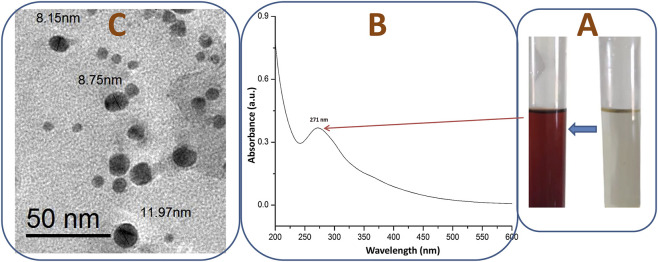
Visual observation **(A)**, UV–VIS spectra **(B)** of biosynthesized SeNPs with FM, and TEM imaging **(C)** of FM-synthesized SeNPs.

The bioreduction/formation of SeNPs *via* FM stabilization/mediation could be recognized visually, as illustrated in Figure 1. The bioreduction process of the mixture composed of FM and sodium selenite solution resulted in a color change from pale yellow to deep orange within 90 min (Figure 1A). UV–VIS spectroscopic analysis of FM-biosynthesized SeNPs confirmed the reduction/transforming into nanoparticle form (Figure 1B), as was evidenced by the λ_max_ value of the reduction solution (271 nm). The TEM images of the FM-mediated SeNPs, with an average diameter of 6.34 nm, verified the formation and dispersion of NPs with presumed spherical shapes (Figure 1C).

Optical characterization verified the high potential of FM to generate SeNPs, which is mainly attributed to the surface plasmon resonance (SPR) of biosynthesized SeNPs, generally reported with a λ_max_ value of approximately 260–275 nm (ElSaied et al., 2021; Youssef et al., 2022). The efficacy of FM in reducing, capturing, and stabilizing SeNPs was confirmed by the appearance of a single UV absorption peak and a deep orange color, indicating successful biosynthesis (Al-Saggaf et al., 2020; El-Sherbiny et al., 2024).

### 3.2 Biochemical interaction assessment via infrared analysis

**FIGURE 2:**
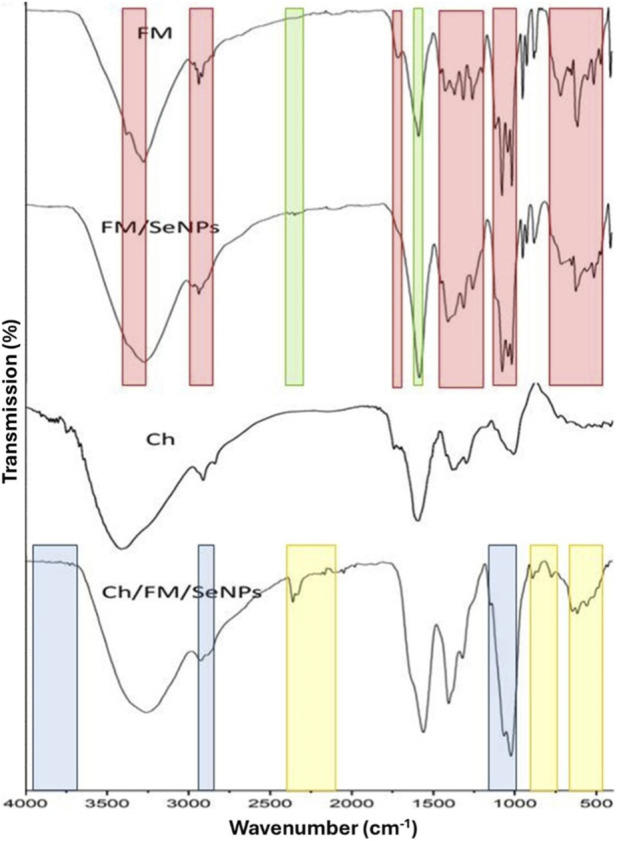
FT-IR spectra for FM, FM/SeNPs, Ch, and Ch/FM/SeNPs.

The FT-IR spectrum, as shown in Figure 2, was used to determine the functional groups/bonds and the interactions involved in the synthesis of SeNPs using FM and the capping of the nanoconjugates using Ch. In the FM spectrum, an absorbance band detected at 3,276 cm^-1^ indicates the presence of hydroxyl groups. Absorption at 2,934 cm^-1^ corresponds to the C-H stretching vibration. Another band occurs at 1,716 cm^-1^, corresponding to the C=O stretching band of the carboxyl (COOH) group. The wavenumbers at approximately 1,591 cm^-1^ and 1,426 cm^-1^ are related to the symmetric stretching vibration of COO^−^. The characteristics bands between 1,200 cm^-1^ and 900 cm^-1^ are identified as the fingerprint region of polysaccharides.

The conjugation and bioreduction of SeNPs with FM resulted in significant adjustments in the IR spectra (Figure 2: FM/SeNPs) compared with the plain FM spectrum. Numerous bands disappeared or showed lower intensity after SeNP reduction (indicated by red highlighted zones), e.g., within the 3,380–3,270 cm^-1^ range, the 2,970–2,850 cm^-1^ range, at 1,716 cm^-1^, within the 1,455–1,190 cm^-1^ range, at 1,120 cm^-1^, and within the 780–470 cm^-1^ range. These changes indicate the involvement of such groups in interactions with Se ions and the breakage of their bonds during NP biosynthesis/reduction. Furthermore, numerous bands emerged/became sharper (indicated by green highlighted zones), e.g., within the 2,380–2,300 cm^-1^ range and at 1,590 cm^-1^, indicating the formation/development of new bonds between FM biomolecules and SeNPs (El Rabey et al., 2024). The clear changes between FM and FM/SeNPs spectra indicate the extent of combination and composition during the biosynthesis of SeNPs using FM, providing strong evidence of biochemical and electrostatic reactions (Gad et al., 2022).

The Ch spectrum (Figure 2, Ch) exhibited the main functional groups/bonds characteristic of standard chitosan (Picos-Corrales et al., 2023). The primary characteristic indicators in the spectrum of plain Ch were observed at approximately 3,414 cm^-1^ (stretching vibration of N–H and O–H groups), 2,909 cm^-1^ and 2,840 cm^-1^ (stretching C–H vibrations), 1,601 cm^-1^ (stretching vibration of NH bonds in amide II), and 1,420 cm^-1^ (CH_2_ bending) (Luque-Alcaraz et al., 2016; Alsharari et al., 2018; Khubiev et al., 2023).

The Ch/FM/SeNP spectrum (Figure 2) indicated numerous biochemical bonds/functional groups transferred from either Ch or FM/SeNP spectra; several derived bands appeared from Ch (indicated by blue highlighted zones), e.g., within the 3,925–3,735 cm^-1^ range, the 2,965–2,855 cm^-1^ range, and the 1,160–990 cm^-1^ range. Additionally, others were derived from FM/SeNPs (indicated by yellow highlighted zones), e.g., within the 2,400–2,100 cm^-1^ range, the 910–735 cm^-1^ range, and the 670–475 cm^-1^ range. These findings may indicate physical, chemical, and electrostatic interactions between both constituents (Ch and FM/SeNPs), likely due to the disparate charges of these components (Santiago-Castillo et al., 2022).

### 3.3 Nanomaterial physiognomies

**FIGURE 3:**
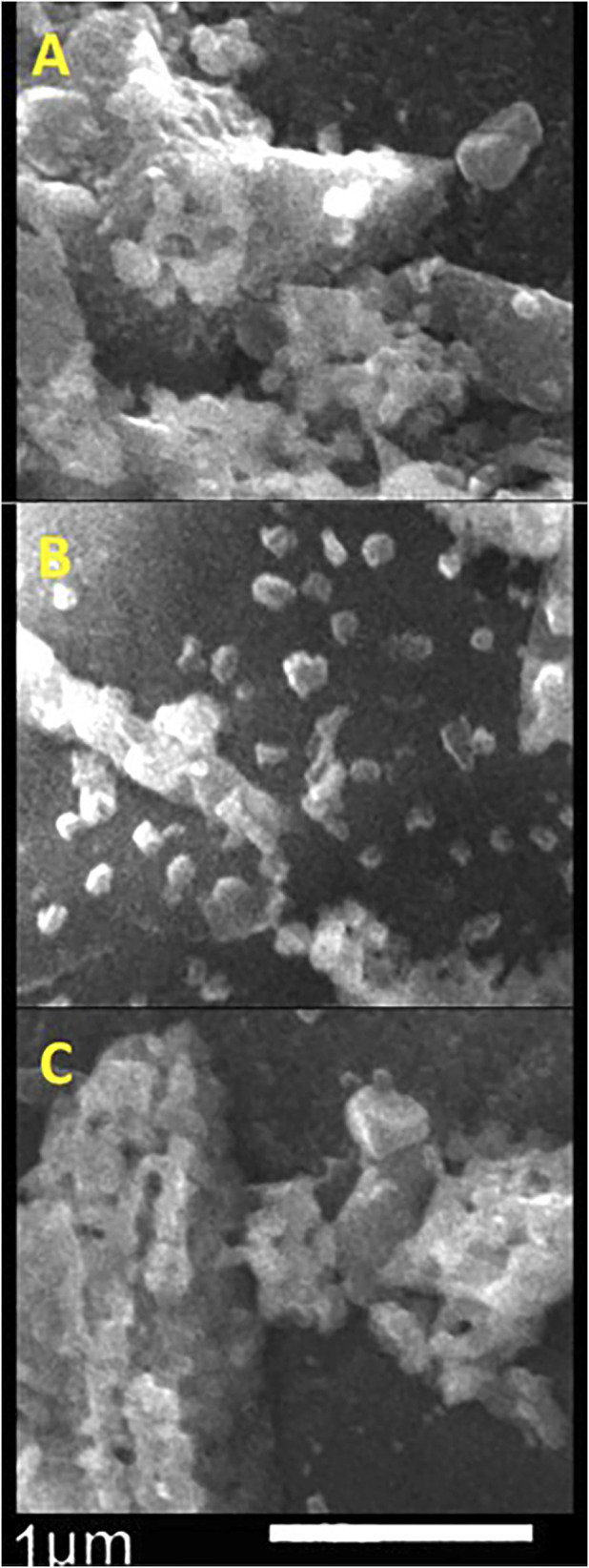
SEM images of the biopolymer nanocomposites: **(A)** T_1_, **(B)** T_2_, and **(C)** T_3_.

The morphological and structural properties of the materials used and the generated NCs were evaluated using DLS analysis (Table 1) and SEM imaging (Figure 3) of the synthesized nanomaterials (Ch/FM/SeNPs NCs; T_1_, T_2_, and T_3_). The charge, size, and distribution of NPs are directly related to DLS pattern evaluation, while the morphological characteristics of the synthesized nanomaterials are displayed by SEM micrographs. As shown in Table 1, DLS measurements obtained using the Zetasizer indicated that Ch had a highly positive ζ potential (+38.21 mV) and an average PS of 78.77 nm, with a size range between 23.11 and 315.53 nm. In contrast, FM/SeNPs exhibited a negative charge (−25.93 mV) with a smaller PS range (2.63–44.94 nm) and an average size of 6.34 nm. Consequently, when Ch was added to FM/SeNPs in T_1_, the charge increased to −13.75 mV. As the ratio of Ch increased in T_2_ then in T_3_, positive charges became dominant on the NCs. At the highest Ch concentration, the ζ potential reached +34.68 mV. The average diameters of T_1_, T_2_, and T_3_ were 96.65, 81.18, and 141.39, respectively. T_2_ exhibited the smallest particle size range (33.93–262.28 nm) and the lowest average particle size (81.18 nm), corresponding to an equivalent quantity (1:1) of Ch and FM/SeNPs. The recorded PDI values for the fabricated nanomaterials/composites indicated the polydispersity of the nanoconjugates. Moreover, SEM images (Figure 3) confirmed the results presented in Table 1 by displaying morphological characteristics indicative of the semi-spherical shapes of the nanocomposites; their average sizes were consistent with those obtained *via* DLS analysis. While the mean size of FM/SeNPs was 6.34 nm, the Ch/FM/SeNPs nanocomposites exhibited larger average sizes, which could be explained by nanoparticle aggregation or polymer encapsulation of the nanoparticles (Aborabu et al., 2024; El Rabey et al., 2024).

**TABLE 1:** Particle size and charge of nanomaterials.

Nanomaterial	Size range (nm)	Average size (nm)	Charge (mV)	PDI
Ch	23.11–315.53	78.77	+38.21	0.58
FM/SeNPs	2.63–44.94	6.34	−25.93	0.42
T_1_ (2 FM/SeNPs: 1 Ch)	41.32–468.67	96.65	−13.75	0.53
T_2_ (1 FM/SeNPs: 1 Ch)	33.93–262.28	81.18	+30.11	0.62
T_3_ (1 FM/SeNPs: 2 Ch)	46.72–493.51	141.39	+34.68	0.60

### 3.4 Acute toxicity and mortality

The selenium and SeNP concentrations determined using ICP were as follows:

453 mg of Se per 1 g of sodium selenite (Na_2_SeO_3_).

4.21 mg of SeNPs per 1 g of FM/SeNP nanocomposite.

2.801 mg of SeNPs per 1 g of Ch/FM/SeNPs (T1 trial).

2.092 mg of SeNPs per 1 g of Ch/FM/SeNPs (T2 trial).

1.401 mg of SeNPs per 1 g of Ch/FM/SeNPs (T3 trial).

The acute oral toxicity study of the Ch/FM/SeNPs nanocomposites trials showed no mortality at the initial doses, and no animal deaths were recorded throughout the duration of the study. These findings indicate the high biosafety of the tested nanocomposites (Zvereva, 2023).

### 3.5 Effect of Ch/FM/SeNPs nanocomposite on body weight

The results showed marked changes in body weight from day 1 to day 28 following diabetes induction in rats (Table 2). The body weight of the diabetic control group was significantly reduced compared to that of the normal control group, which is a common complication associated with diabetes. The mean body weight of GI (normal control group) increased from an initial mean of 162.74 ± 2.11 to 205.16 ± 3.87 after 28 days. In contrast, GII (diabetic control group) showed a reduction in body weight from 158.81 ± 8.46 to 138 ± 7.32, and the reduction in body weight in diabetic rats may indicate metabolic abnormalities leading to tissue degradation and muscle weakness. The disruption of pancreatic β-cells by diabetes eventually leads to physico-metabolic disorders, including reduced bodyweight gain (Pwaniyibo et al., 2020). However, treatment with Ch/FM/SeNPs nanocomposite reduced diabetes-related weight loss in the treated groups compared to the diabetic control group (GII). The reduction in weight loss observed with different nanocomposite doses was comparable to the effect of glibenclamide (a standard antidiabetic drug). Body weight is a sensitive indicator of the health status of experimental animals, and a reduction in body weight reflects metabolic impairment within the body (Abdallah et al., 2017; Ibikunle et al., 2022).

**TABLE 2:** Effect of Ch/FM/SeNPs nanocomposite on body weight of STZ-induced diabetic rats.

Group	Day 0	Day 14	Day 28
Normal control (GI)	162.74 ± 2.11	179 ± 3.34	205.16 ± 3.87
Diabetic control (GII)	158.81 ± 8.46	151 ± 8.37	138 ± 7.32
Treated 1 (GIII)	160.49 ± 5.87	165 ± 4.65	185 ± 4.42
Treated 2 (GIV)	165.87 ± 6.79	176 ± 5.84	193 ± 5.21
Treated 3 (GV)	169.31 ± 2.89	181 ± 3.58	197 ± 3.34
Glibenclamide (GVI)	167.96 ± 3.34	180 ± 3.61	195 ± 3.3.46

### 3.6 Effect of Ch/FM/SeNPs nanocomposite on blood glucose levels

The results indicated that the blood glucose level in the diabetic group was significantly higher (327.74 ± 7.56 mg/dl) than that in the normal control group (88.53 ± 1.23 mg/dl) during the experimental period (days 0–28). A marked reduction in blood glucose levels was observed in the diabetic groups treated with the Ch/FM/SeNPs nanocomposite (GIII, GIV, and GV), comparable to that observed in the glibenclamide-treated group (GVI). The blood glucose levels were 216.67 ± 7.27 mg/dl, 146.12 ± 6.03 mg/dl, 112.54 ± 3.68 mg/dl, and 110.26 ± 3.76 mg/dl, respectively. The glibenclamide-treated group (GVI) exhibited the lowest blood glucose level (110.26 ± 3.76 mg/dl). Notably, the Ch/FM/SeNPs nanocomposite-treated group receiving 0.3 mg/kg showed a significantly greater reduction in blood glucose levels compared to the groups receiving 0.1 and 0.2 mg/kg (Table 3).

**TABLE 3:** Effect of Ch/FM/SeNPs nanocomposite on blood glucose levels in STZ-induced diabetic rats.

Groups	Day 0	Day 14	Day 28
Normal control (GI)	91.03 ± 2.37	89.38 ± 1.78	88.53 ± 1.23
Diabetic control (GII)	382 ± 6.77	357 ± 6.87	327.74 ± 7.56
Treated 1 (GIII)	365 ± 5.64	251.93 ± 4.42	216.67 ± 7.27
Treated 2 (GIV)	346 ± 5.32	226.76 ± 5.51	146.12 ± 6.03
Treated 3 (GV)	316 ± 6.71	187.31 ± 4.15	112.54 ± 3.68
Glibenclamide (GVI)	314 ± 6.15	183 ± 3.87	110.26 ± 3.76

Many research reports have demonstrated the antihyperglycemic activity of OFI fruit mucilage in STZ-induced hyperglycemic rats, which has been attributed to the presence of flavonoids and phenolic compounds responsible for its antidiabetic activity. This activity may be associated with the inhibition of the enzymes α-glucosidase and α-amylase, both of which are involved in carbohydrate breakdown, consequently decreasing blood glucose levels (Mohammed et al., 2025; Juárez-Flores et al., 2025). Flavonoids and phenolic compounds have also been reported to influence glucose metabolism through various mechanisms, such as inhibiting carbohydrate digestion and glucose absorption in the intestine, stimulating insulin secretion by pancreatic β-cells, regenerating damaged β-cells in diabetic rats, acting as insulin secretagogues, moderating hepatic glucose release, activating insulin receptors, and enhancing hepatic glucose utilization (Zhao et al., 2020; Hussain et al., 2022; Guan and Zhang, 2025).

Moreover, some studies have shown that Se may also play a role in controlling the redox status of pancreatic β-cells through the production of antioxidant selenoproteins and by participating in the protection of β-islet cell membranes against oxidative damage, thereby restoring normal β-cell mass and insulin secretion (Karalis, 2019; Yang et al., 2021; Ghazwani et al., 2025). Interestingly, research findings on SeNPs have reported a significant reduction in hyperglycemia, possibly by promoting insulin-mimetic activity, increasing insulin secretion and liver glycogen content, enhancing the activity of glucose-6-phosphate dehydrogenase, and reversing the abnormal activity of certain gluconeogenic and glycolytic liver enzymes (Khalil et al., 2024; Das et al., 2025). In many cases, hypoglycemic activity and Se content are positively correlated; it has been reported and confirmed that increasing Se content contributes to higher hypoglycemic activity of Se-containing polysaccharides (Das et al., 2025). Thus, the synergistic action of SeNPs and Ch in reducing hyperglycemia is presumably due to their antioxidant activity, β-cell protection, or improved insulin sensitivity.

As glibenclamide is a sulfonylurea used to treat type 2 diabetes by stimulating pancreatic beta cells to increase insulin secretion, primarily by binding to and inhibiting ATP-sensitive potassium (K_ATP_) channels, the action of the nanocomposites in the present study was comparable to that of this drug, indicating similar mechanisms of action for the fabricated nanocomposites (Zeng et al., 2018; Abozaid et al., 2023).

### 3.7 Effect of Ch/FM/SeNPs nanocomposite on lipid profile

According to Table 4, compared with the normal control group, the diabetic control group showed significantly elevated levels of total cholesterol (TC), triglycerides (TG) and low-density lipoproteins (LDL), along with decreased levels of high-density lipoproteins (HDLs). Gradually, the levels of TC, TG, and LDL were significantly reduced, whereas HDL levels were significantly increased in the Ch/FM/SeNPs nanocomposite-treated groups (0.1, 0.2, and 0.3 mg/kg). The anti-hyperlipidemic effect observed in the glibenclamide-treated group was comparable to that recorded in Ch/FM/SeNPs nanocomposite-treated group receiving 0.3 mg/kg.

**TABLE 4:** Effect of Ch/FM/SeNPs nanocomposite on lipid profile of STZ-induced diabetic rats.

Groups	Total cholesterol mg/dl	Triglycerides mg/dl	HDL cholesterol mg/dl	LDL cholesterol mg/dl
Normal control (GI)	97.18 ± 2.78	76.38 ± 2.44	44.75 ± 1.15	37.05 ± 1.17
Diabetic control (GII)	182.40 ± 2.57	171.58 ± 3.24	19.67 ± 0.73	124.31 ± 3.21
Treated 1 (GIII)	133.21 ± 2.48	89.45 ± 3.03	34.77 ± 1.37	75.62 ± 2.75
Treated 2 (GIV)	125.40 ± 1.79	83.12 ± 2.13	39.65 ± 1.52	58.40 ± 2.44
Treated 3 (GV)	113.28 ± 0.76	79.23 ± 0.67	48.22 ± 1.07	38.14 ± 2.38
Glibenclamide (GVI)	108.13 ± 1.64	84.30 ± 2.52	46.42 ± 1.12	41.25 ± 2.34

These results were in agreement with several previous studies reporting that hypercholesterolemia, hypertriglyceridemia, increased LDL levels, and reduced HDL levels were commonly observed in diabetes, possibly due to the stimulation of lipolysis in adipose tissue, which causes hyperlipidemia and increases the risk of heart disease and stroke (Khouloud et al., 2017; Elshehy et al., 2020). The Ch/FM/SeNPs nanocomposite-treated groups showed results closest to those of the normal control group in all tests and exhibited lower serum levels of TC, TG, and LDL, along with higher serum HDL levels, compared with the diabetic group. As previously reported, OFI and SeNPs have significant hypolipidemic effects and contribute to improvements in the lipid profile (Díaz et al., 2017; Huang et al., 2020; Li et al., 2023). These results suggest that the Ch/FM/SeNPs nanocomposite, similar to glibenclamide, may be used either as a standalone treatment or in combination with conventional antidiabetic therapies to reduce the potential risk of cardiovascular disease.

### 3.8 Effect of Ch/FM/SeNPs nanocomposite on liver and kidney function

The serum alanine aminotransferase (ALT), aspartate aminotransferase (AST), creatinine, and urea levels were significantly elevated in the diabetic control group compared with the normal control group. However, treatment with different doses of the Ch/FM/SeNPs nanocomposite and glibenclamide (5 mg/kg) reversed the STZ-induced changes, as shown in Table 5. Normal ALT levels averaged approximately 45.24 U/L, whereas the diabetic group exhibited an average level of 56.83 U/L. Glibenclamide reduced the average ALT level to approximately 49.31 U/L, while the Ch/FM/SeNPs nanocomposite (0.1, 0.2, and 0.3 mg/kg) showed average ALT levels of approximately 50.47 U/L, 47.87 U/L, 46.24 U/L, respectively. Normal AST levels averaged approximately 66.13 U/L, compared with 89.11 U/L in the diabetic group. The average AST level was approximately 68.47 U/L in the glibenclamide-treated group and 81.78 U/L, 72.50 U/L, and 65.71 U/L in the Ch/FM/SeNPs nanocomposite-treated groups (0.1, 0.2, and 0.3 mg/kg), respectively.

**TABLE 5:** Effect of the Ch/FM/SeNPs nanocomposite on hepatic and renal parameters of STZ-induced diabetic rats.

Groups	ALT (U/L)	AST (U/L)	Creatinine (mg/dl)	Urea (mg/dl)
Normal control (GI)	45.24 ± 2.31	66.13 ± 3.02	0.52 ± 0.04	19.83 ± 2.02
Diabetic control (GII)	56.83 ± 3.28	89.11 ± 2.45	1.32 ± 0.16	37.24 ± 3.16
Treated 1 (GIII)	50.47 ± 2.46	81.78 ± 1.18	0.97 ± 0.08	31.25 ± 2.84
Treated 2 (GIV)	47.87 ± 2.21	72.50 ± 1.23	0.82 ± 0.07	26.49 ± 2.48
Treated 3 (GV)	46.24 ± 2.04	65.71 ± 1.37	0.70 ± 07	23.20 ± 1.97
Glibenclamide (GVI)	49.31 ± 1.88	68.47 ± 2.63	0.69 ± 0.06	21.14 ± 1.89

The diabetic group showed increased creatinine (60.6%) and urea (46.75%) levels compared with the normal control group. The Ch/FM/SeNPs nanocomposite-treated groups (0.1, 0.2, and 0.3 mg/kg) showed reductions in creatinine levels of 26.5%, 37.87%, and 46.96%, respectively and reductions in urea levels of 16.08%, 28.86%, and 37.70%, respectively, compared with the diabetic group. The glibenclamide-treated group showed reductions in creatinine and urea levels of 47.72% and 43.23%, respectively, compared with the diabetic group.

The liver is the primary organ responsible for maintaining blood glucose levels within specific limits, in addition to its vital role in the storage, detoxification, metabolism, and excretion of xenobiotics and their metabolites. Elevated activities of serum aminotransferases (ALT and AST) are common indicators of liver diseases and diabetes (Dadey et al., 2026). Transaminases are highly sensitive biomarkers, and increased levels have been closely linked to impaired hepatic function, hepatic damage, and toxicity (Nouri et al., 2020; Das et al., 2025; Dadey et al., 2026). Interestingly, studies by Steinbrenner et al. (2022) suggested that Se may play a role in controlling the redox state of pancreatic β-cells by enhancing the synthesis of antioxidant selenoproteins, such as GPx, and helping protect the cell membranes of β-islets from oxidative damage, thereby promoting the restoration of normal β-cell mass and insulin secretion. Furthermore, according to Das et al. (2025), Se supplementation increased the levels of other important antioxidants (including SOD, CAT, and GSH). According to Khater et al. (2021), the significant elevation of liver and kidney function markers (ALT, AST, creatinine, and urea) was strongly correlated with oxidative stress and the depletion of antioxidant enzymes (SOD and GPx). The Ch/FM/SeNPs nanocomposite showed potential hepatoprotective properties, particularly in comparison with the effects of diabetes on the liver. This effect may be attributed to the protective action of the mucilage, which reverses the damaging effects of diabetes through its antioxidant properties. Diabetic hyperglycemia induces elevation in serum creatinine and urea levels, which are considered significant markers of kidney dysfunction and damage. The present results indicate that Ch/FM/SeNPs nanocomposite improved impaired renal function associated with diabetic nephropathy. It has been reported that Ch-stabilized SeNPs have potential roles in ameliorating hepatic damage/apoptosis, insulin deficiency, and cardiac impairment associated with type 2 diabetes, mainly through the regulation of the caspase, Fas/FasL, and Bax/Bcl-2 pathways (Mohamed et al., 2021). Additionally, Ch/SeNPs conjugates exhibited beneficial effects against diabetic nephropathy in a rat model of “type 2 diabetes.” Ch/SeNPs reduced the prevalence of TNF-α, IL-1β, and IL-6 following diabetes induction and effectively protected the kidney function while reducing renal tissue injury through the prevention of oxidative stress and restoration of glucose hemostasis (Khater et al., 2021). Furthermore, oral treatment with Ch/SeNPs resulted in significant improvements in insulin levels, blood glucose, ALT, AST, IGF-1, and CK-MB compared with STZ-induced diabetic rats; Ch/SeNPs composites also significantly decreased the expression levels of the CAPN10 and TCF7L2 genes while increasing the expression of the PPAR-γ gene (Abozaid et al., 2023).

Although the study lasted 28 days, which could suggest the potential for adverse effects due to selenium accumulation and toxicity, no mortality or abnormal consequences were observed in the treated animals. This may be attributed to the rational doses of SeNPs in the fabricated nanocomposites, their biosynthesis using natural materials, and their encapsulation within Ch biopolymers, which have been reported to reduce the potential toxicity of SeNPs. Furthermore, no specific toxic effects were identified that could be solely attributed to selenium (Ryabova et al., 2024).

With the innovative fabrication of Ch/FM/SeNPs nanocomposites and their promising action as an antidiabetic formulation, it can be inferred that the synergistic action of all nanocomposite components contributes to enhancing resistance against diabetic complications in an STZ-induced diabetic rat model. The scale-up of mucilage-based SeNP synthesis for pharmaceutical applications appears highly feasible as the plant materials are readily available and cost-effective worldwide, and the applied synthesis protocol is simple, facile, uncomplicated, and does not require high costs or energy input.

## 4 Conclusion

The SeNPs were successfully biosynthesized using FM into stable particles with a small size (6.34 nm) and homogeneous distribution. The Ch/FM/SeNPs nanocomposites were innovatively fabricated and characterized; their antidiabetic effects were evaluated in an STZ-induced diabetic rat model. Significant improvements were observed in body weight, blood glucose levels, lipid profile, and liver and kidney function tests in the diabetic groups treated with Ch/FM/SeNPs nanocomposites compared with the diabetic control group. The Ch/FM/SeNPs nanocomposite powder at a concentration of 0.3 mg/kg was found to reduce the severity of hyperglycemia and showed promising results in improving diabetes-related indicators. Based on the findings of this study, the use of Ch/FM/SeNPs nanocomposite represents a promising approach for managing/controlling hyperglycemia in diabetes patients and may serve as an effective selenium-based drug candidate for the treatment of DM. Given the well-documented sex-based differences in diabetes pathophysiology and insulin sensitivity, further research incorporating both sexes is warranted to fully understand the potential sex-related differences in the observed effects. One limitation of the current study is the exclusive use of male Wistar rats. Although this approach was chosen to avoid the confounding influence of the estrous cycle on metabolic and hormonal variables, it may limit the generalizability of our findings to females. Further characterization of the nanomaterials using additional analytical techniques (e.g., XRD and Raman spectroscopy) is recommended to confirm crystallinity and bonding characteristics. Furthermore, the evaluation of oxidative stress markers (e.g., MDA, SOD, and GPx) is recommended to strengthen mechanistic insights.

## Data Availability

The original contributions presented in the study are included in the article/supplementary material; further inquiries can be directed to the corresponding authors.
